# Complete Nucleotide Analysis of the Structural Genome of the Infectious Bronchitis Virus Strain Md27 Reveals its Mosaic Nature

**DOI:** 10.3390/v1031166

**Published:** 2009-12-04

**Authors:** Arun Ammayappan, Vikram N. Vakharia

**Affiliations:** 1 Center of Marine Biotechnology, Biotechnology Institute, University of Maryland, 701 E. Pratt Street, Baltimore, MD 21202, USA; E-Mail: ammayapp@umbi.umd.edu (A.A.); 2 Department of Veterinary Medicine, University of Maryland, College Park, MD 20742, USA

**Keywords:** infectious bronchitis virus, structural genes, sequencing, phylogenetic analysis, recombination

## Abstract

Infectious bronchitis virus (IBV) causes highly contagious respiratory or urogenital tract diseases in chickens. The Maryland 27(Md27) strain was first isolated in 1976 from diseased chicken flocks in the Delmarva Peninsula region. To understand the genetic diversity and phylogenetic relationship of existing strains with Md27, the complete nucleotide sequence of the 3′end coding region (∼7.2 kb) of Md27 was determined and compared with other IBV strains and coronaviruses. It has the same S-3-M-5-N-3′ gene order, as is the case of other IBV strains. The spike gene of Md27 exhibits 97% identity with the SE17 strain. There are deletions at the spike gene, non-coding region between M and 5 genes, and at the 3′ untranslated region (UTR), which is different from Ark-like strains. Phylogenetic analysis and sequence alignments demonstrate that Md27 is a chimera containing different gene segments that are most closely related to the SE17, Conn and JMK strains. This current study provides evidence for genomic mutations and intergenic recombination that have taken place in the evolution of IBV strain Md27.

## 1. Introduction

Infectious bronchitis virus (IBV), a member of the *Coronaviridae*, order *Nidovirales,* is a pathogen of domestic chickens that causes acute, highly contagious respiratory disease [1,2]. The IBV genome contains a single, positive-strand RNA molecule, which is about 27.6 kb long and has a cap at the 5′end and poly (A) tail at the 3′end [3]. It comprises ten open reading frames (ORFs) and the first 20 kb genome is made up of ORF1, which is a replicase gene. The replicase has two ORFs, 1a and 1b [4]; among which 1b is produced as 1ab polyprotein by a -1 ribosomal frame-shifting mechanism [4]. The ORF1 encodes non-structural proteins associated with RNA replication and transcription. The IBV genome codes for four major structural proteins; the spike (S) glycoprotein, the small envelope (E) protein, the membrane (M) glycoprotein, and the nucleocapsid (N) protein [5]. In addition to this, IBV has other genes that encode for non-structural proteins interspersed among structural genes, namely 3a, 3b, 5a and 5b [6].

Numerous IBV serotypes, such as Arkansas, Massachusetts, Connecticut, Florida, Georgia and others, referred to as variants, exist in the United States of America (USA). These variants have practical significance for controlling the disease because immunity following infection or vaccination with one serotype often is not protective against subsequent infections with heterologous serotypes [7,8]. Many serotypes have been described for IBV, probably due to the frequent point mutations that occur in RNA viruses and also due to recombination events [9–11]. It is essential to characterize field isolates for the selection of appropriate vaccine strains.

The late Dr. Warren Marquardt (Department of Veterinary Medicine, University of Maryland) received samples from poultry diagnostic laboratories on the Delmarva Peninsula from 1971 through 1974 [12]. Out of 106 isolates made, three were not neutralized by any kind of serum. Two of these appear to be identical on the basis of a serum neutralization test and had been designated as Maryland 27 (Md27). The Md27 antiserum has an unusually broad spectrum of minor cross-reactions with the other viruses [12]. The Md27 strain has never been characterized by sequencing and it is essential to sequence these typical strains to understand the evolution of IBV geographically and also to implement an effective vaccination program to control new variant IBV strains. Here, we describe the complete sequence analysis of the 7.27 kb of 3′end of the genome of Md27 IBV strain and its comparison and phylogenetic relationship with many heterologous IBV strains, and other coronaviruses from different parts of the world.

## 2. Results and Discussion

The structural organization of Md27 and nucleotide identity with other IBV strains is shown in Tables 1 and 2, respectively. The spike protein gene of Md27 is 3507 nucleotides long (1168aa) and codes for a protein of approximately 128.6 kDa. The transcription regulating sequence (TRS) CTTAACAAA is located 49 nucleotides from the start codon of the spike gene. The spike protein is most likely glycosylated and has 22 potential N-glycosylation sites (Asn-Xaa-Ser/Thr) which are predicted by NetNGlyc 1.0 server (http://www.cbs.dtu.dk/services/NetNGlyc/). Hydrophobicity analysis (http://www.cbs.dtu.dk/services/TMHMM) predicted the occurrence of one transmembrane (TM) domain at (1100–1122aa) in the C-terminus of the spike protein and one endodomain (1122–1168aa). The Spike protein is cleaved into S1 and S2, of which S1 produces neutralizing and serotype specific antibodies [13,14]. The spike protein cleavage site for Md27 (His-Arg-Ser-Arg-Arg/Ser) is located between residues 544 and 545. The S1 protein is closely related to the SE17 strain (96% identity) and exhibits <86% identity with other IBV strains. The S2 gene is more conserved than S1 and has 98% identity with Ark DPI, Gray, Jilin, JMK and SE17 strains. The S2 subunit may also induce serotype specific neutralizing antibodies and S2 subunits are conserved within a serotype but not between serotypes [15]. There is a notable deletion of 3 nucleotides in the S1 gene between nucleotides 69 and 70, which is different from other Ark-like strains.

Gene 3 codes for two non-structural proteins (3a and 3b) and one structural protein (E), which is a small membrane protein of coronavirus. Gene 3a and 3b start at the last nucleotide of the S gene and 3a genes, respectively. Gene 3b overlaps 3c by 20 nucleotides and 3a has a signal sequence at the very 5′ end (nucleotides 1–22). The small membrane protein E, encoded by a 107 nucleotides long ORF, is approximately 12 kDa in size. A TRS (CTGAACAAT) is located 20 nucleotides upstream of the ATG codon. Gene 3a of Md27 virus shows 97% identity with Conn, Cal99 and Ark99 strains. The 3b gene has 98% identity with Ark DPI, Ark99, Cal99, Conn, HK, and Jilin strains, whereas gene E shows 98% identity with Ark DPI, Conn, HK and Jilin. The comparative sequence alignment indicates that the E protein gene is more conserved than the genes coding for 3a and 3b.

Gene 4 codes for the matrix (M) protein of 223aa, which is the most abundant envelope glycoprotein of coronaviruses, and it overlaps the E gene by 23 nucleotides. Its molecular mass depends on the glycosylation and varies from 25 to 33 kDa [16]. The M protein of Md27 contains a single putative N-linked glycosylation site at amino acid position 4. The M protein of coronaviruses differs in glycosylation types. In case of IBV it is N-linked, whereas in MHV, it is O-linked [13,17]. Gene M of Md27 shows 99% identity with Ark DPI, Conn, HK and Jilin. RNA 5 of Md27 is dicistronic and it encodes for two nonstructural proteins, 5a and 5b, respectively.

There is a 350 nucleotides long non-coding region between gene M and 5 with a deletion of 9 nucleotides, which is similar to Mass-type IBV strains. Gene 5 is more conserved than any of the other genes of Md27, and it is nearly identical (98–99%) to other strains.

RNA 6, the smallest but the most abundant RNA in IBV-infected cells, encodes for the N protein. The N protein is the only phosphorylated structural protein in coronaviruses [18]. The N protein has many basic residues, and serine residues accounting for 8 to 10% of the total amino acids in the N protein. The abundance of serine residues accounts for the specific phosphorylation of serine residues [19,20]. The N protein of Md27 contains 33 serine residues. It has 96% identity with Ark DPI, Ark99, CK/CH/LSD/05I, Conn, HK and Jilin strains.

**Figure 1.**
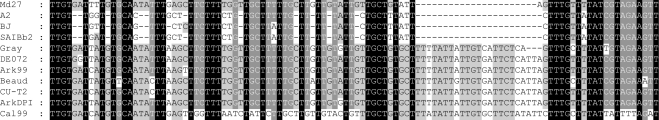
Comparison of 5′ terminus of 3′ untranslated region (UTR) of Md27 with some selected strains of IBV*. Nucleotide deletion of Md27 at 3′UTR is clearly shown. Sequences were compared using Gendoc software. *Nucleotides of Md27 shown are starting from 86^th^ nucleotide after the stop codon of N gene.

The Md27 has a 483 nucleotides long 3′UTR. Interestingly, there is a deletion of 22 nucleotides in the 5′ terminus of 3′UTR (Figure 1), which shows some similarity to the Chinese strains A2, BJ and SAIBb2. In the case of IBV, C- and N-terminal regions of the N protein (but not the central region) interact with the 3′end of the non-coding region of IBV genomic RNA [21]. This deletion suggests possible involvement of 3′UTR in replication, which may influence pathogenesis of the virus. The 3′UTR of Md27 is closely related (97–98% identity) to Ark DPI, DE072, Beaudette, Gray, M41, Jilin and CU-T2. The 3′UTR (∼161nucleotides) immediately downstream of the N gene is U-rich and it is a highly variable region of the coronavirus genome. It has been observed that the 3′ UTR is involved in genome replication of coronaviruses, despite its apparent ability to possess quite variable sequence and sequence lengths [22].

**Figure 2.**
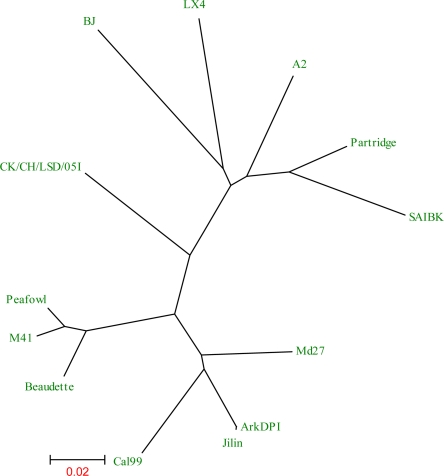
Comparison of 7.27 kb of the 3′end of the Md27 genome sequence with other IBV strains by an unrooted phylogenetic tree. Sequences are aligned by neighbor-joining method using MEGA4 program. Md27 clustered with ArkDPI, Jilin and Cal99 IBV strains. The scale at the bottom indicates the number of substitution events.

**Figure 3.**
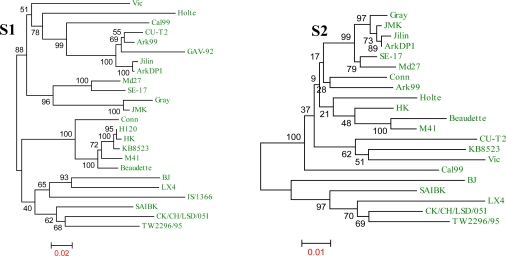

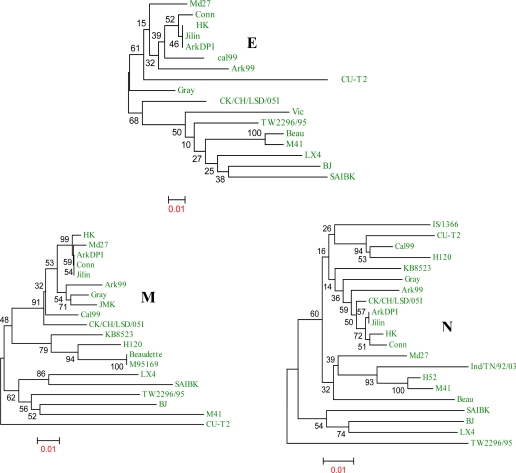
Phylogenetic analyses of the structural genes of Md27 virus with those of other IBV strains. The Md27 strain had clustered with ArkDPI, HK, Conn, and Cal99 in most of the trees. The tree is constructed from aligned nucleotide sequences of structural genes (S1, S2, spike gene; E, envelope gene; M, matrix gene; N, nucleocapsid gene) by neighbor-joining method with 1000 bootstrap replicates using the MEGA4 program. For each tree, the bootstrap value for each branch is indicated and the scale at the bottom indicates the number of substitution events.

The phylogenetic relationship of specific genes of the Md27 with other IBV strains is illustrated in Figures 2 and 3. Comparisons are made for the structural genes (S1, S2, E, M and N) because nucleotide differences between strains mainly occur in these regions [23–26] The S1 and S2 gene of Md27 clusters with the SE-17 strain and in the E and M gene phylogeny it clusters with Ark DPI, Jilin, Conn, and HK. In the N gene phylogeny Md27 clusters with HK, Conn, Ind/TN/92/03 and H52 (Figure 3). Comparison of the structural genes of Md27 with other coronaviruses demonstrates high sequence identity with turkey coronavirus (TCoV) and IBVs from peafowl and partridge (Table 3). Among the four structural genes, M is highly conserved between coronaviruses, whereas the E protein is less conserved.

**Figure 4.**
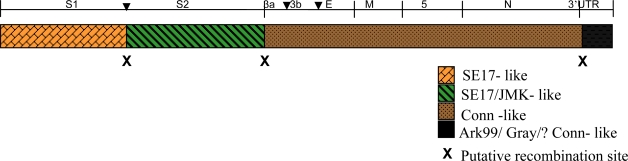
Schematic representation of the structural genome of Md27. Entire structural genome of Md27 was analyzed for its similarity with other IBV strains. Structural genes and their ORFs are marked by ▾. Putative recombination sites in Md27 genome are denoted by **x**. Picture is not drawn to scale.

The complete structural genome analysis of Md27 suggests that it is a chimera of virus genomes represented by the SE17, Conn, JMK and Ark 99 IBV strains (see Figure 4), which were circulating during that time period [12,27]. The S1 and S2 genes of Md27 exhibit 96% and 98% identity, respectively, with SE17. Most of the nucleotide differences between Md27 and SE17 are in the S1 gene, especially in the hypervariable regions (HVR). The remainder of the structural sequence is not available for SE17, so it is difficult to conclude the genetic relationship of other genes of Md27 with SE17. Based on the limited data available (as many other strains are not yet sequenced), it is conceivable that the spike gene is derived from SE17 by recombination.

The Md27 shares close homology (96–99%) with the Conn strain, starting from gene 3a to the end of the N gene, which suggests that most of the Md27 structural genome was derived from a Conn strain. Table 2 shows the comparison of the entire 7 kb genome of Md27, which reveals that Ark DPI and Jilin share high levels of sequence identity with Md27. Earlier we reported that Jilin is actually an Ark DPI strain [28]. Ark DPI was isolated during the 1980s [27], and it was demonstrated that Ark DPI is a direct derivative of the Conn strain [28]. This study suggests that Md27 and Ark DPI are derived from the same ancestor, but diverged independently by recombination with different strains. Several factors determine IBV evolution, mainly high mutation rate due to the absence of proof-reading mechanisms, and recombination between strains because of widespread use of live vaccines, immunological pressure and frequent mixed infections [29–31]. Our data has provided the evidence that both genomic mutations and intergenic recombination have taken place in the evolution of Md27 IBV strain and its descendents.

## 3. Materials and Methods

### 3.1. Virus

Virus isolation was done in Dr. Marquardt’s laboratory by passaging tissue (trachea or kidney) homogenates into 9–10-day-old chicken embryos by the allantoic cavity route of inoculation and at least three passages were done before declaring samples as negative. Two of the isolates were named as Md27, and these were propagated by inoculating into 9–day-old embryonated specific-pathogen-free (SPF) chicken eggs and allantoic fluid was collected 72 h post-inoculation. The fluid was clarified by low speed centrifugation and clear supernatant was stored at −80 °C until further use.

### 3.2. RNA extraction and amplification

Genomic RNA was extracted from virus-infected allantoic fluid with Qiagen (Valencia, CA, USA) RNAeasy kit, following the manufacturer’s instructions and stored at −80 °C until further use. Oligonucleotides were designed based on the Ark DPI11 sequence (GenBank accession No. EU418976). Overlapping primers were designed in a manner such that each pair of primer covered approximately 2 kb of the genome. All gene fragments were amplified using RT-PCR kit (Invitrogen, Carlsbad, CA, USA), according to the manufacturer’s instructions and the RT-PCR products were cloned into the pCR2.1 TOPO TA^®^ vector (Invitrogen, CA). To determine 3′-terminus of Md27 genomic RNA, reverse transcription was carried out with a reverse poly (T) primer (5′-GCGGCCGCTTTTTTTTTTTTTTTT TT-3′) and an internal gene specific forward primer.

### 3.3. DNA sequence analysis

DNA from various clones was sequenced by dideoxy chain termination method using an automated DNA sequencer (Applied Biosystems Inc., Foster City, CA). At least three independent clones were sequenced for each amplicon to exclude errors that can occur from RT and PCR reactions. The assembly of contiguous sequences was performed with the GeneDoc software [32]. Comparative sequence analyses of MD27 with other IBVs and coronaviruses were conducted using the BLAST search, NCBI, and Vector NTI Advance 10. Phylogenetic analyses were carried out using the MEGA4 program [33]. The phylogenetic tree was constructed from aligned nucleotide and amino acid sequences using the neighbor-joining method with 1000 bootstraps.

### 3.4. GenBank accession numbers

The GenBank accession number for the MD27 sequence is FJ008695. The accession numbers for other IBV gene sequences used in this study are as follows: (a) complete structural genomes: TCoVMG10, NC_010800; Beaudette, NC_001451; CK/CH/LSD/05I, EU637854; A2, EU526388; Peafowl/GD/KQ6/2003, AY641576; Cal99, AY514485; M41, DQ834384; partridge/GD/S14/2003, AY646283; BJ, AY319651; LX4, AY338732; SAIBK, DQ288927; HK, AY761141; Vic, DQ490221; KB8523, M21515; TW2296/95, DQ646404 (b) S1: Jilin, AY839144; Gray, L18989; Holte, L18988; UK/2/91, Z83976; Qu16, AF349620; JMK, L14070; H52, AF352315; H120, M21970; GAV-92, AF094817; DE072, AF274435; IS/1366, EU350550; (c) S2: JMK, AF239982; Jilin, AY839146; Holte, AF334685; DE072, AY024337; Conn, AF094818; Gray, AF394180; H120, AF239982; (d) S: Ark99, L10384; CU-T2, U04739; (e) Gene 3: Jilin, AY846833; Conn, AY942752; CU-T2, U46036; Ark99, AY942751; Gray, AF318282 (f) M: Jilin, AY846833; JMK, AF363608; H120, AY028295; Gray, AF363607; (g) Gene 5: Jilin, AY839142; Gray, AF469011; DE072, AF203000; (h) N: Jilin, AY839145; Ind/TN/92/03, EF185916; H52, AF352310; H120, AY028296; Gray, M85245; (i) 3′UTR: H120, AJ278336.

## 4. Conclusions

In this study we analyzed ∼7.2 kb of the 3′ genomic end of the IBV strain Md27 and compared it with other IBV strains and coronaviruses from different parts of the world. This analysis suggests that Md27 evolved by recombination of different strains which were circulating during the same time period. This study demonstrates that recombination is the major evolutionary mechanism for infectious bronchitis virus to create new strains and variants.

## Figures and Tables

**Table 1.** Structural genome organization of Md27*.

**Gene**	**Frame**	**Start**	**Stop**	**Size (nucleotides)**	**Size (amino acids)**
S	+1	1	3507	3507	1168
3a	+3	3507	3680	174	57
3b	+2	3680	3874	195	64
3c(E)	+3	3855	4178	324	107
M	+1	4156	4827	672	223
5a	+3	5178	5375	198	65
5b	+2	5372	5620	249	82
N	+1	5563	6792	1230	409
3′ UTR		6793	7276	483	

* The first nucleotide of start codon of spike gene is counted as nucleotide 1 of the structural genome.

**Table 2.** Percent (%) identity of Md27 structural genes with other IBV strains.

**IBV Strains**	**S1**	**S2**	**3a**	**3b**	**3c(E)**	**M**	**5a**	**5b**	**N**	**3′UTR**	7kb*
ArkDPI	86(84)	**98(98)**	91	**98**	**98(96)**	**99(98)**	**99**	**98**	**96(96)**	**98**	**95**
Ark99	85(84)	**96(95)**	**97**	**98**	**95(91)**	**97(96)**	NA	NA	**96(95)**	**96**	NA
Beaudette	83(80)	94(93)	86	84	88(82)	91(93)	93	**96**	93(94)	**97**	91
BJ	77(76)	85(89)	86	76	87(81)	90(93)	86	93	89(91)	88	85
Cal99	82(79)	94(93)	**97**	**98**	93(88)	**96(95)**	**99**	**98**	94(95)	90	91
CK/CH/LSD/05I	78(75)	87(90)	85	84	92(87)	**96(96)**	**99**	**99**	**96(96)**	91	89
Connecticut	83(80)	**95(96)**	**97**	**98**	**98(97)**	**99(98)**	**99**	**98**	**96(95)**	NA	NA
CU-T2	85(83)	94(93)	94	97	91(87)	88(86)	**98**	**98**	94(93)	**97**	NA
DE072	62(50)	75(76)	NA	NA	NA	NA	**98**	**98**	NA	**98**	NA
Gray	86(82)	**98(97)**	NA	NA	**96**(92)	**97(97)**	**98**	**98**	95(95)	**97**	NA
GAV-92	83(79)	NA	NA	NA	NA	NA	NA	NA	NA	NA	NA
H120	83(80)	NA	NA	NA	NA	91(94)	NA	NA	92(94)	82	NA
H52	83(80)	NA	NA	NA	NA	NA	NA	NA	95(95)	NA	NA
HK	83(80)	**95(95)**	91	**98**	**98(95)**	**99(98)**	**99**	**98**	**96(95)**	44	NA
Holte	83(78)	94(94)	NA	NA	NA	NA	NA	NA	NA	NA	NA
Ind/TN/92/03	NA	NA	NA	NA	NA	NA	NA	NA	94(94)	NA	NA
IS/1366	77(74)	NA	NA	NA	NA	NA	NA	NA	92(94)	NA	NA
Jilin	86(84)	**98(98)**	91	**98**	**98(96)**	**99(98)**	**99**	**98**	**96(96)**	**98**	95
JMK	86(84)	**98(98)**	NA	NA	NA	**97(96)**	NA	NA	NA	NA	NA
KB8523	83(80)	90(91)	NA	NA	NA	93(94)	NA	NA	93(95)	NA	NA
LX4	76(75)	85(88)	81	76	89(81)	90(91)	83	91	88(91)	NA	26
M41	83(79)	94(94)	84	85	88(82)	91(94)	90	96	95(94)	97	91
Qu16	84(81)	NA	NA	NA	NA	NA	NA	NA	NA	NA	NA
SAIBK	81(79)	87(90)	82	82	85(81)	89(92)	85	**96**	87(92)	90	86
SAIBb2	NA	NA	NA	NA	NA	NA	NA	NA	NA	85	NA
SE 17	**96(93)**	**98(98)**	NA	NA	NA	NA	NA	NA	NA	NA	NA
TW2296/95	80(78)	86(89)	81	85	88(83)	90(91)	86	95	88(89)	NA	NA
UK/2/91	77(75)	NA	NA	NA	NA	NA	NA	NA	NA	NA	NA
Vic	82(79)	89(91)	84	88	88(87)	89(94)	88	94	90(93)	NA	NA

Sequences with >95% identity are indicated in bold letters; Amino acid sequences identity are within the parenthesis; NA-Not available for Analysis.

* Entire structural genome, starting from start codon of spike gene to last nucleotide of IBV genome.

**Table 3.** Percent (%) amino acid identity of Md27 structural proteins to other coronaviruses^a^.

**Coronaviruses^b^**	**S**	**E**	**M**	**N**
BatCoV	21	11	29	23
BCoV	21	13	30	24
ECoV	21	13	30	24
FCoV	26	16	23	22
HCoV 229E	26	10	25	22
TGEV	26	20	25	25
MHV A59	22	14	31	25
Peafowl, Avian	**86**	**82**	**94**	**95**
Partridge	**84**	**81**	**92**	**92**
SARS CoV	20	16	29	23
SW1	25	28	35	34
TCoV	33	**91**	**97**	**94**

a Sequences with >50% identity are in bold letters;

b BatCoV, Bat coronavirus; FCoV, feline coronavirus; HCoV, human coronavirus; BCoV, bovine coronavirus; MHV, mouse hepatitis virus; SARS-CoV, severe acute respiratory syndrome coronavirus; Partridge, Avian infectious bronchitis virus partridge/GD/S14/2003, Peafowl, Avian infectious bronchitis virus isolate Peafowl/GD/KQ6/2003, SW1, beluga whale coronavirus; TCoV, turkey coronavirus.
